# The community ecology perspective of omics data

**DOI:** 10.1186/s40168-022-01423-8

**Published:** 2022-12-13

**Authors:** Stephanie D. Jurburg, François Buscot, Antonis Chatzinotas, Narendrakumar M. Chaudhari, Adam T. Clark, Magda Garbowski, Matthias Grenié, Erik F. Y. Hom, Canan Karakoç, Susanne Marr, Steffen Neumann, Mika Tarkka, Nicole M. van Dam, Alexander Weinhold, Anna Heintz-Buschart

**Affiliations:** 1 Department of Environmental Microbiology, Helmholtz Centre for Environmental Research – UFZ, Leipzig, Germany; 2 German Centre for Integrative Biodiversity Research (iDiv) Halle-Jena-Leipzig, Leipzig, Germany; 3 Institute of Biology, Leipzig University, Leipzig, Germany; 4 Department of Soil Ecology, Helmholtz Centre for Environmental Research- UFZ, Halle, Germany; 5 Institute of Biodiversity, Friedrich Schiller University, Jena, Germany; 6 Institute of Biology, University of Graz, Graz, Austria; 7 Department of Botany, University of Wyoming, Wyoming, USA; 8 Department of Biology and Center for Biodiversity and Conservation Research, University of Mississippi, Oxford, Mississippi USA; 9 Department of Biology, Indiana University, Indiana, USA; 10 Institute of Biology, Geobotany and Botanical Garden, Martin Luther University Halle Wittenberg, Halle, Germany; 11 Leibniz Institute of Plant Biochemistry, Bioinformatics and Scientific Data, Halle, Germany; 12 Leibniz Institute of Vegetable and Ornamental Crops (IGZ), Großbeeren, Germany; 13 Swammerdam Institute for Life Sciences, University of Amsterdam, Amsterdam, Netherlands

**Keywords:** Multivariate statistics, Molecular ecology, Community ecology

## Abstract

The measurement of uncharacterized pools of biological molecules through techniques such as metabarcoding, metagenomics, metatranscriptomics, metabolomics, and metaproteomics produces large, multivariate datasets. Analyses of these datasets have successfully been borrowed from community ecology to characterize the molecular diversity of samples (*ɑ*-diversity) and to assess how these profiles change in response to experimental treatments or across gradients (*β*-diversity). However, sample preparation and data collection methods generate biases and noise which confound molecular diversity estimates and require special attention. Here, we examine how technical biases and noise that are introduced into multivariate molecular data affect the estimation of the components of diversity (i.e., total number of different molecular species, or entities; total number of molecules; and the abundance distribution of molecular entities). We then explore under which conditions these biases affect the measurement of *ɑ*- and *β*-diversity and highlight how novel methods commonly used in community ecology can be adopted to improve the interpretation and integration of multivariate molecular data.

Video Abstract

**Supplementary information:**

**Supplementary information** accompanies this paper at 10.1186/s40168-022-01423-8.

*One of the most fundamental patterns of scientific discovery is the revolution in thought that accompanies a new body of data* [1].

## Introduction

The direct characterization and analysis of sampled pools of biomolecules, particularly DNA, RNA, proteins, and metabolites, has fundamentally altered the life sciences and specifically microbiology. Metagenomics and metabarcoding can identify organisms in a sample and detect inter- and intraspecific diversity, while (meta-)transcriptomics, (meta-)proteomics, and metabolomics can characterize the functional responses of biological individuals, populations, or whole communities (Table 1). Despite their different targets, these techniques measure molecular entities in a high-throughput, high-data volume manner and produce similar multivariate data outputs, enabling multivariate, molecular ecology (heretofore MME).

**Table 1:** MME techniques yield data sets with common structures, and often, limitations

	Common techniques	0-inflated	No N	Compositional
Genomics: The system-wide identification and quantification of DNA sequences and the encoded functions in an organism or population [2].	High throughput sequencing		(+)	
Transcriptomics: The system-wide identification and quantification of the RNA transcripts in an organism or population [3].	High throughput sequencing, microarrays	+	+	+
Proteomics: The use of quantitative protein-level measurements of gene translation to characterize biological processes and decipher the mechanisms of gene expression control [4].	Mass spectrometry	+	+	
Metabolomics: The systematic identification and quantification of metabolites (small molecule substrates, intermediates, products of cell metabolism) in an organism or population [5]..	Nuclear magnetic resonance spectroscopy, mass spectrometry	+	(+)	
Metabarcoding: The large-scale identification and quantification of variation of diversity in an environmental sample in terms of a specific genomic region (DNA) [6].	High throughput amplicon sequencing	++	+	+
Metagenomics: Large-scale identification and quantification of all DNA in an environmental sample [7].	High throughput shotgun metagenomic sequencing	++		+
Metatranscriptomics: Large-scale identification and quantification of all RNA transcripts in an environmental sample [8]	High throughput RNA sequencing, (microarrays)	++		+
Metaproteomics: Large-scale identification and quantification of the entire protein complement from an environmental sample [9].	Mass spectrometry	++	+	
Meta-metabolomics: Large-scale identification and quantification of small molecules from an environmental sample [10].	Nuclear magnetic resonance spectroscopy, mass spectrometry	+	+	

For techniques with “no N,” the total number of molecular entities measured contains no biological information. For techniques that produce 0-inflated datasets, the data matrices contain more zeros than non-zero values, while for compositional datasets, the abundance of species is correlated to the technique and contains no biological information. For each limitation, whether it is an issue or a serious issue for each data type is indicated with + or ++, respectively. Data types that face a limitation but it seldom affects the scientific questions asked with these data are indicated with (+)

MME techniques measure a wide range of molecular entities within a selected class of molecules*.* Being generally untargeted, they require less *a priori* knowledge about the biomolecules measured than targeted assays. Interest in MME techniques has grown rapidly over the past two decades, and their application has informed ecological and evolutionary theory. For example, metabarcoding has been used to show how dormancy affects the spatial distribution of microbes [11], and metatranscriptomic analyses have revealed that microbial niche differentiation in the rhizosphere is both spatially and temporally regulated [12]. Similarly, a combination of proteomics and targeted metagenomics have been used to study adaptation in light of horizontal gene transfer [13].

MME data and conventional community ecology (CE) data are both multivariate and the *sample × molecule* matrices produced by MME techniques may be viewed as analogous to the *plot × species* matrices produced by community ecologists. Like in community ecology, studies in molecular and microbial ecology are often less concerned with the response of single molecular entities, especially when the identity and function of specific molecular entities are unknown. Rather, they focus on differences or changes in a whole profile or on emergent properties of the community (e.g., metabolic cooperation). Similar to CE studies, MME data are often used to characterize molecular diversity and the relative abundance of molecular entities within samples and across space, time, or experimental treatments.

**Fig. 1:**
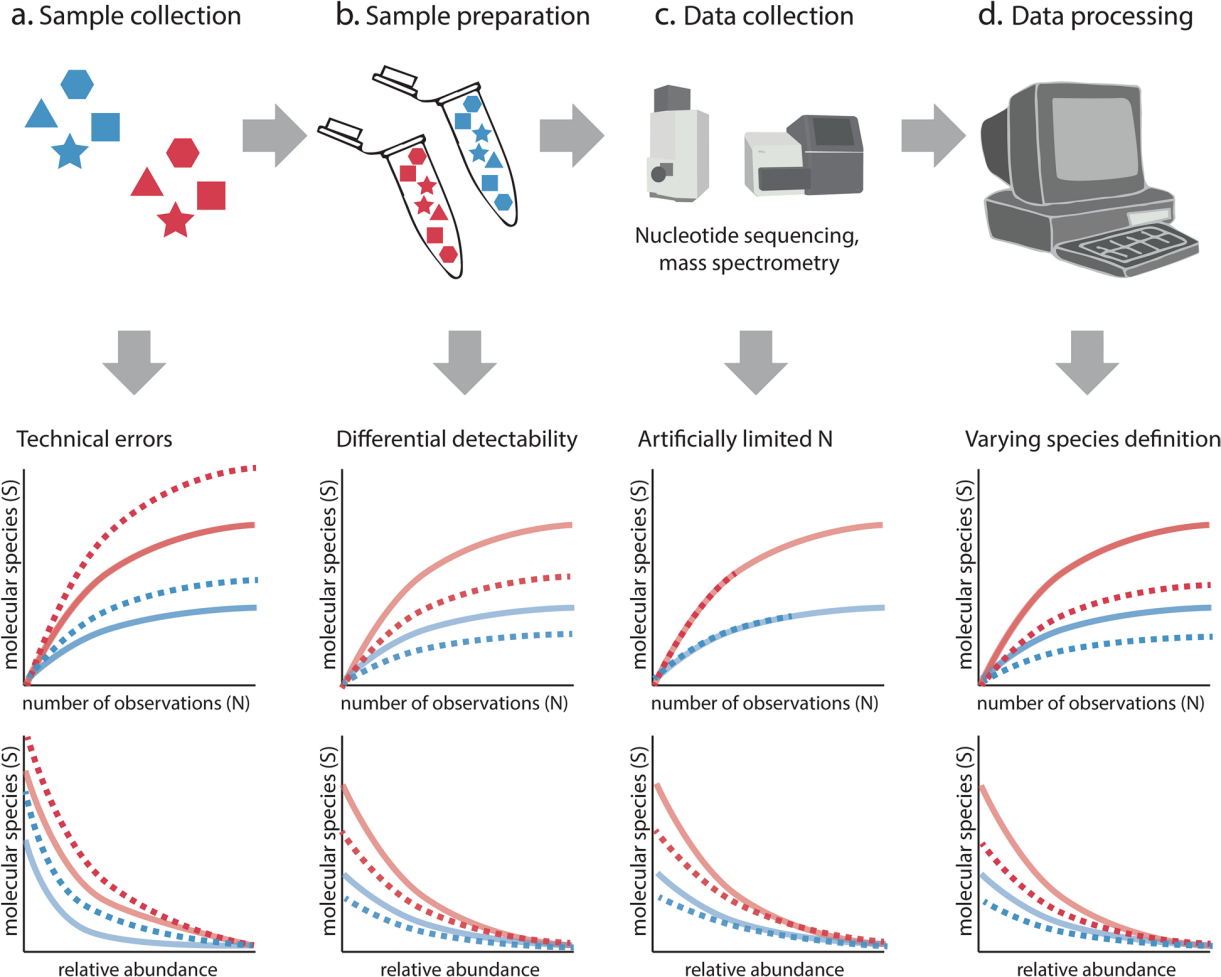
Sample collection and preparation, data collection, and post-processing are inextricably linked in MME techniques and can all potentially affect estimates of diversity. The effect of researcher choices on S, N (middle row), and SAD (bottom row) during data generation is shown below each step. The true diversity in two samples is shown in red and blue, and the measured diversity is shown as dotted lines. Technical errors during sample collection and storage can increase S (e.g., due to non-specific contamination [19]), resulting in higher estimates for S and steeper SAD (**a**). In contrast, sample preparation can reduce the detectability of certain molecular entities (e.g., during PCR amplification in metabarcoding [20]) resulting in a lower S and flatter SAD (**b**). Technical limitations on the number of observations are imposed by some of the data collection instruments used (e.g., sequencer), placing technical limits on N, and potentially resulting in more even communities (i.e., a flatter SAD) (**c**). During processing, applying a less stringent species definition can result in reduced S and a flatter SAD (**d**)

ɑ-and β-diversity metrics, which describe species diversity in a sample and the difference in diversity between samples, respectively, were developed by CE and are often used to describe changes in the molecular profiles of samples (e.g., Shannon diversity indices, Bray-Curtis dissimilarities; [14–16]). In CE, diversity metrics rely on three key measures [17, 18] (Fig. 1): the absolute numbers of individuals detected in a specific area (N), the total number of species in that area or species richness (S), and the relative abundances of community members (i.e., species abundance distributions, or SAD).

CE measures are affected by biases that are introduced during the molecular sample preparation, molecular analyses, data collection, and processing steps that generate MME data. The techniques that produce MME data are relatively new, and while much work has been devoted to identifying their biases and limitations, how these affect the applicability of CE measures to assess patterns of molecular diversity has received less attention. However, within the context of ecology, the biases and limitations of diversity data have been extensively discussed, resulting in various approaches to overcome them. For example, issues related to detection limits given finite sample sizes are potentially the oldest analytical research topic in ecology [21] and have led to the development of methods that can quantify sample completeness (i.e., rarefaction, diversity index estimators [22–24]). Importantly, methods from molecular data analysis and ecology can be used to analyze different kinds of MME data with their specific challenges (Table 1), but they make different assumptions about the underlying distribution of the data [25]. Consequently, their applicability depends both on the raw data and the scientific questions [26].

 Here, we explore how detection biases and technical limitations associated with MME data affect the calculation of N, S, and SAD, and how this, in turn, affects the determination of ɑ- and β-diversity. Finally, drawing parallels from other areas of community ecology, we indicate analytical approaches that allow for the robust estimation of diversity metrics for biased or limited MME data.

## The components of biodiversity in the context of MME data

### Selection of sampling scale and species definition

All components of biodiversity—and the patterns emerging from them—depend on the sampling scale [27, 28]. There is no single “perfect” scale which is optimal for measuring biodiversity, and the relative importance of different ecological processes is intricately linked to the scale of observation [29]. However, defining the scale of observation in MME analyses is not straightforward, and altering this scale is not always possible. First, the data collection method used (e.g., mass spectrometer, sequencer) dictates how samples must be prepared and indirectly sets limits on the scale of sampling. In low biomass samples, the lower limits can be hard to achieve, leading to biases due to contaminants or artifacts [19]. On the other hand, upper limits can be very small (e.g., μL of a liquid or mg of a solid), so sample preparation must be adapted for the analytical sample to adequately represent the desired scale. Second, during each step of sample preparation, the substrate obtained in the previous procedure is often subsampled, which may further dissociate diversity assessments from the initial sampling scale. Third, sampling strategies that are common in MME techniques can have poorly defined scales (e.g., filtrate, swabs), which complicates the comparison diversity in samples obtained with even slight variations in sampling approaches. Incomparability of sampling scales cannot always be avoided (e.g., if body sites or plant tissues are being compared [30, 31]). The mismatch between the scale of observation and the scale at which the target organisms perceive and function in their environment has received substantial attention in ecology [28, 32] and has recently been discussed in the context of microbes [33], but further research into the spatial scale of influence on MME target molecules is necessary.

The spatial scaling of diversity has been explored in metabarcoding data [34–37], but further research is urgently needed to describe the spatial components of complex samples used for other kinds of MME data, while explicitly acknowledging that scaling up observations is not always possible for MME data and may be constrained by the system of interest (e.g., host-associated systems) and by the technique used (i.e., the laboratory protocols). Additional research is also needed to determine whether small sample sizes limit the ability to detect phenomena that occur at larger scales or that depend on larger organisms [38].

In order to capture microbial diversity at larger scales and reduce the effect of small-scale spatial variability, pooling (i.e., homogenizing multiple smaller samples from a larger area) is a common practice [39]. However, the universal distance decay relationship (DDR, [40]) predicts that samples taken further away from each other in space and time will have less overlap in species, and this is also likely the case for MME data (e.g., sequence-based omics [41, 42]). The spatial distribution of pooled samples can therefore greatly affect (and when unstandardized, bias) diversity estimates [38], resulting in inflated diversity estimates for samples which were pooled from larger areas. Furthermore, MME techniques can target molecules from a wide range of organisms of varying body sizes, but body size affects the distribution of diversity [43–45]. Further research is needed to determine to what extent the DDR applies to MME data (e.g. ecometabolomics of larger organisms), and whether this has implications for sampling designs. For microbial communities, the relative similarity of functional compared to taxonomic profile has been demonstrated [46, 47], but whether DDRs are observable in functional microbial MME data has not been analyzed to date.

While MME techniques often impose limits on the sampling scale, they generally require the researcher to explicitly select a definition of the units of diversity. These units can be species or molecular entities, such as ASVs or protein families. If the definition is not given (as it is, e.g., for identified metabolites), it is usually based on a threshold of molecular similarity of detected molecules to each other or to references. Definitions vary with e.g., 97 to 100% similarity for metabarcoding [48], 95% average nucleotide identity for microbial genomes [49, 50], and a vast range of identity and expected value thresholds for functional units, such as gene families. Importantly, the choice of units and definition directly affects biodiversity measurements (Fig. 1). Within the context of synthesis, archiving raw MME data allows data reusers to reprocess datasets using a single-species definition, allowing cross-study comparisons.

### The number of individuals (N)

In CE, for a given sampling scale, N measures the number of individuals in that space. However, MME techniques measure molecules rather than individuals. The two are not always related) e.g., because of differences in cell or body sizes, or in copy numbers of marker genes). Furthermore, the number of detected molecules often reflects a machine’s throughput, rather than biological reality [51]. For example, in sequencing-based methods (i.e., (meta-)genomics and (meta-)transcriptomics), the number of observations or sequencing reads per sample (i.e., sequencing depth) reflects the choice of sequencer and the number of samples loaded, rather than the abundance of organisms in the sample [52]. As a consequence for MME data, N often serves only as an indicator of observation effort (e.g., sequencing depth) and is otherwise uninformative.

The decoupling of N and the abundance of molecules in situ creates two limitations. First, it precludes the estimation of the true abundances of molecular entities. Second, uneven observation depths make changes in the abundances of molecular entities (i.e., differential detection) sensitive to the normalization method used [53]. Several statistical approaches have been developed to normalize before detecting changes in molecular entities across samples through generalized linear models, including DESeq2 [54], metagenomeSeq [55], edgeR [56], LEfSE [57], and voom [58]. However, several normalization methods assume similar SADs and they can bias the comparison of abundances across samples (e.g., in transcriptomic data; [53]). Given the wide range of options, the development of novel tools for comparing workflows and the resulting MME data (e.g., ANPELA in proteomics [59]) and studies comparing different approaches for differential detection [25, 60, 61] are increasingly important. For example, in LC-MS and metabolomics data, spectral data are normalized by external standards, pooled samples, or total biomass [62].

### Species richness (S)

CE’s species richness in an area (S) can be equated to the number of distinguishable molecular entities in a sample (i.e., molecular richness), which are often defined *ad hoc*, as discussed above. S can be influenced by the environment, dispersal, interactions among organisms, or changes in organisms producing molecules of interest (as in [17]). Assessing the number of molecular entities present is often of interest, with multiple studies having found connections between richness, mechanistic processes, and ecological phenomena. For example, metabarcoding-based assessments of soil bacterial communities revealed that bacterial richness was positively related to carbon decomposition and soil enzymatic activities [63]. Similarly, LC-MS-based assessments of the secondary metabolites of fungi revealed species-specific metabolic richness [64].

The detectability of molecular entities is not evenly distributed (Fig. 1), and this may negatively affect S (and SAD, see below). For all MME techniques, several hundred to thousands of molecular entities may be present in concentrations that span multiple orders of magnitude, which complicates their measurement. Nonrandom differences in the detectability of molecular entities may lead to the consistent underestimation of specific molecular entities, as is the case with biases caused by different primer affinities in metabarcoding [65], or to the underestimation of molecular entities below a certain abundance threshold. For example, the limited dynamic range of a mass spectrometer may cause rare molecular entities to fall below the limit of detection, while very abundant entities may saturate the detector, pushing them above the limit of quantification [66]. Differences in detectability can also be random and result in lower signal-to-noise ratios (e.g., in proteomics data [67]). Mathematical modeling of persistent detection biases has been proposed as a first step to identify where biases arise and to quantify them in metagenomic data [68], while latent variable modeling has been proposed for estimating missing values in proteomics data [69].

### Species abundance distributions (SAD)

In CE, species abundance distributions (SAD) describe how abundances vary across species in a community (often expressed as “relative abundances,” standardized by total biomass or the total number of individuals in the community [70]). SAD of all ecological communities exhibit a hollow shape, as some species are more abundant than others, but the shape can vary [70]. Flatter SAD indicate a more even community, while hollower SAD indicates strong dominance by certain species. Like community data, MME data generally have few very abundant molecular entities and many with low abundances (e.g., in metabarcoding [71]).

Because SAD are distributions rather than a single metric, comparing SAD across multiple samples is not straightforward, as differences in the relative distribution of taxa (i.e., evenness) can result in different relationships between samples depending on N [72]. This is important for the analysis of SAD, since N in MME tends to relate to technical choices rather than biological reality, and the different numbers of molecules per molecular species further confound the estimation of the number of organisms in the sample [38]. Nevertheless, N affects the other components of biodiversity: the greater the observation effort, the greater S will be, and the greater the number of rare molecular entities that will be found (i.e., a longer-tailed SAD).

When the number of observations is artificially determined by the technique (i.e., uninformative N), an increase in one species may result in an observed decrease of another, even if the absolute abundance of the other molecular entity is unchanged [73]. This limitation makes the data compositional [74], so the observed abundance of each species depends on the abundance of all other species in the sample [52], skewing SAD. Several methods to analyze compositional data [74–77], identify differentially abundant molecular entities [78, 79] or groups of molecular entities [51], and determine causality [80] have been proposed. In the most basic form, compositional analyses use log ratio transformations to individual values or the geometric mean of all values (clr). These were applied, for example, to metabarcoding and metalomic data [81]. However, MME data are often sparse (i.e., molecular entities appear seldom across samples) and zero-inflated (i.e., there are more zeros than expected from the distribution of the observed molecular entities in a sample, Table 1). Because of the large number of zeros, log transformations and ratios do not work [82]. Overcoming this limitation remains an area of active research: the simplest approach to removing zeros is replacing zeros with fixed, low values that lie below the instrument’s detection limit. However, this skews sparse data further [73]. Alternatively, methods that test pairwise ratios of specific molecular entities remove rare features (e.g., ANCOM [83], ANCOM-II [84]). More complex methods use Bayesian inference, but are computationally expensive (i.e., ALDEX2 [85]). For metabolomic data, testing the informative potential across all possible ratios of metabolite pairs is a common and statistically validated practice [86]. To model the relationship between molecular entities and environmental gradients, generalized joint attribute models adapt joint species distribution models to zero-inflated data [87] and are appropriate for MME data.

For many MME questions, rare molecular entities are uninformative, in addition to being more likely observed due to technical error, making them less quantifiable. MME fields have developed different approaches to remove such data. In untargeted metabolomics, molecular entities that are detected in blanks or exhibit high variation across technical replicates are removed from the metabolomic profile [62, 88]. In most modern metabarcoding pipelines, molecules that appear only once are assumed to be indistinguishable from sequencing errors and are removed automatically [89–91], artificially shrinking S [92], N, and resulting in a flatter SAD. However, this may be preferable to including erroneous data. For example, one study found that at most, 40% of molecular entities that appeared once (i.e., singletons) could potentially be artefactual, directly affecting species richness estimation [93]. In many analysis pipelines, rare taxa are commonly filtered to reduce the dimensionality of the data, regardless of the “correctness” of the observation. This is especially true for differential detection methods, which require a minimum abundance to detect differences among samples (e.g., ANCOM-II and DESeq2; [54, 84]).

How SAD curves are used in CE to derive multiple diversity estimates, including evenness and ɑ-diversity for any N, as well as to compare abundance profiles across gradients [94] is discussed below.

## Derived diversity components and diversity metrics for MME data

The challenge of analyzing “imperfect” MME data is mirrored by similar issues that commonly arise in CE studies [95]. For example, data from plant or intertidal rock communities are often estimated in terms of “relative cover” (that is, the area covered by each species, divided by the total area covered by any organism). The resulting data are often subject to high error rates, risk undercounting the abundance of rare species, and introduce major statistical challenges like zero inflation [96, 97]. Historically, these problems have severely limited the kinds of analyses that can be applied using cover data. Modern statistical methods partially address them, for example by separately tracking trends in abundance vs. presence/absence data [98], or by modeling the sources of zeroes (e.g., “true” absences vs. “false” absences; [99]).

### Estimating the true N, S, and SAD

Issues related to detection limits given finite sample sizes are one of the oldest analytical research topics in ecology [21]. Rarefaction or diversity index estimators can help quantify how complete a particular sample is likely to be and, in some cases, to standardize data across different samples [22, 23]. These are commonly applied to metabarcoding and other sequence-based MME data [100], especially when the aim is to quantify diversity profiles. However, data loss associated with rarefaction remains controversial [101]. More recent methods for extrapolating diversity estimates based on abundance or presence/absence community data [24, 102] allow for the estimation of diversity in MME data without massive losses in data.

In CE, detection errors that result in missing data are often estimated by revisiting a site (i.e., in vegetation surveys), with marked capture-recapture experimental designs, or with *a posteriori* modeling [103]. Several approaches approximate the true S for DNA-based omics (reviewed in [104]), but such approximations have received less attention from other MME methods. Assuming that rare molecular entities (i.e., singletons) represent technical errors produced during sample processing and data collection [105], targeted methods can estimate the true diversity of these rare molecular entities based on the frequencies of more abundant entities [106, 107]. Similarly, diversity estimators such as the jackknife or Chao1 [108] derive an asymptotic diversity estimate based on the number or of singleton or doubleton entities.

Species occupancy models may be used to correct for false positive and false negative errors [109–112] and approximate the true S and SAD, but when the probability of detection is low, species occupancy models require large numbers of technical and biological replicates [113], which are often not possible. Approaches such as joint species distribution modeling and analyses of species-environment associations [114, 115] can be applied to predict the identities of potential missing species and to introduce* post hoc* corrections to incomplete samples. However, this approach draws heavily on *a priori* understanding about species’ natural histories as well as historical data from previous studies, and may become more prominent as the molecular world is increasingly well characterized.

For the estimation of SAD, parametric curves [70] can improve relative abundance estimates. From the histogram of observed relative abundances, different classical models can be fit (reviewed in [70]). The best fitting model can be selected and then compared across samples. The parameters estimated from curve-fitting, such as Fisher’s *ɑ*, can serve as a diversity metric that reflects the imbalance between few dominant species versus many rare species. For example, a community with a few highly abundant species and many rare ones will show a low ɑ value compared to a similarly rich community with more even abundances. Fisher’s *ɑ* is more sensitive to species of medium abundances and thus more helpful in the context of incomplete sampling [116].

Due to the compositional nature of MME data, less abundant or less detectable components can be pushed below detection thresholds limits which is akin to the classic ecological notion of “veil lines” [21]. Post hoc statistical corrections such as species distribution models attempt to reconstruct information about missing molecular entities based on the observed composition of local or regional species pools [117].

### ɑ-diversity

**Fig. 2:**
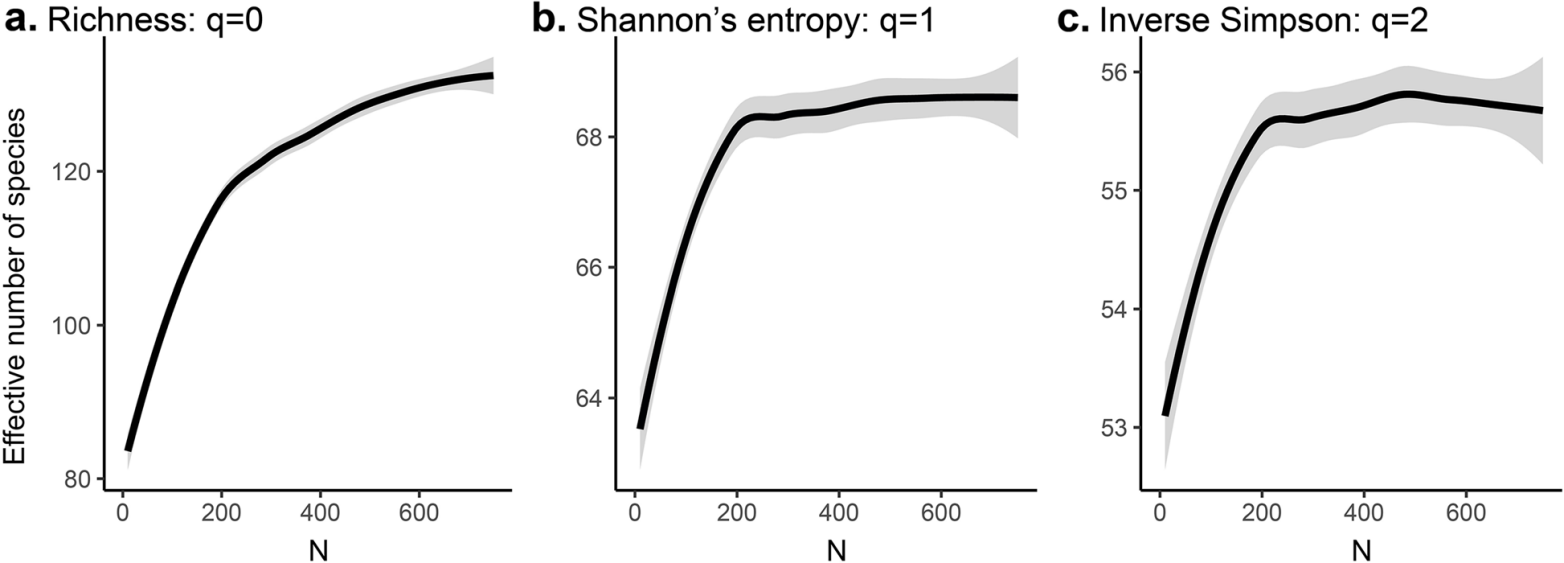
Measuring ɑ-diversity with Hill numbers. *ɑ*-diversity indices were first unified by ecologist Mark Hill as the inverse of mean proportional abundances in a community [123, 124]. The value of *q* (or order of diversity) describes how this mean is calculated, affecting the sensitivity of diversity indices to rare species. In **a**–**c**, Hill numbers are shown for a metabarcoding data obtained from the fecal sample of an Ecuadorian finch (publicly available in NCBI with accession number SRR6486665 [125]). When *q*=0, the weighted harmonic mean of species’ proportional abundances is measured, and richness is assessed (**a**). When *q*=1, the weighted geometric mean is measured, and Shannon’s entropy is assessed (**b**). When *q*=2, the weighted arithmetic mean is measured and inverse Simpson’s richness is assessed (**c**). All Hill numbers are expressed in units of effective numbers of species, or the number of species that would be expected in a community in which all species are equally abundant

Because of the long tail of rare molecular entities and the variable, uninformative N, computing a spectrum of diversity indices can provide a more complete picture of the diversity profile of a sample [118, 119]. In particular, Hill numbers go beyond single-point diversity estimators for fairer comparison between samples across different observation depths [120], leveraging the accumulation of individuals to produce standardized, continuous diversity estimators (Fig. 2). This continuum of estimates is obtained by varying the exponent *q* (or order) of the Hill numbers. As *q* increases, the relative importance of abundant species increases, providing information on whether common or rare species contribute most to *ɑ*-diversity. Hill numbers have been shown to be more robust diversity estimators from molecular data, especially with *q*>1, as they are less sensitive to rare species and sparse datasets [121]. Importantly, because MME data processing and treatments disproportionately affect rare molecular entity, higher order Hill numbers can more robustly estimate *ɑ*-diversity regardless of the researcher’s technical decision-making [122]. An added advantage of Hill numbers is that they can produce confidence intervals along the accumulation of samples, quantifying uncertainty clearly compared to point estimates [24]. Leveraging Hill numbers of *q*>1 is particularly useful for datasets in which there is a high variation in S across samples (e.g., metabarcoding, metagenomics) and in which the community is not fully characterized [119]. It is important to note, however, that the increased robustness of higher order Hill numbers does result in the loss of information about rare species.

### β-diversity

*β*-diversity metrics are commonly used across MME data to quantify the extent to which the molecular entities observed differ between samples. In general, *β*-diversity is assessed by using similarity or dissimilarity metrics, and the choice of metric depends on the data’s limitations as well as the research question of interest. For example, in the commonly used metric Euclidean distances (e.g., in principal components analyses), species absences are equally as informative as presences, making them unsuitable for characteristically sparse MME datasets [126]. In contrast, the semimetric Bray-Curtis dissimilarities give more weight to mutual presences and do not consider mutual absences among two samples to be informative.

If the emphasis is on the presence and absence of molecular entities rather than on their relative abundances, MME data can be converted into incidence data, and Sorensen dissimilarities can be calculated [126]. Importantly, incidence-based assessments are heavily affected by rare molecular entities, whose detection (or non-detection) may be artifactual in the case of MME data [127]. Filtering can also bias the measurement of *β*-diversity in molecular data. For example, in metabarcoding, the practice of removing rare species has recently been shown to artificially decrease *α*- and increase *β*-diversity, while reducing the discriminatory power of *β*-diversity [128].

Similar to *α*-diversity, *β*-diversity indices based on Hill numbers have been developed [129, 130], and allow adjusting the relative importance of rare versus abundant species when computing β-diversity through the *q* parameter. For example, when comparing the similarity between two communities, it is possible to give more weight to shared abundant species than to shared rare species. By varying the value of *q,* these *β*-diversity assessments can draw continuous diversity profiles. As *q* increases, the removal of rare species becomes less central to the calculation of *β*-diversity.

By permuting the available data to create a random or null expectation of the distributions of entities in a community, null modeling-based approaches can distinguish between changes in S and SAD for a wide range of dissimilarity metrics [131, 132]. Incidence-based *β*-diversity metrics can be decomposed into nestedness, which quantifies the extent to which samples with smaller numbers of species are subsets of more species-rich samples, and turnover, which quantifies the replacement, or difference of species between samples [133]. Partitioning *β*-diversity changes can shed light into the ecological processes driving molecular diversity (e.g., in metabarcoding [134]).

When hierarchical information on the similarity among molecular entities is available (e.g., in metabarcoding [135] and proteomics data [136]), phylogenetic *β*-diversity indices can be used to estimate how the relatedness among molecular entities affects observed community changes [137, 138]. The interpretation of variation in phylogenetic diversity indices can help tease apart evolutionary mechanisms at play [139], reviewed in [140]. For example, phylogenetic clustering can be the result of habitat filtering or of a constrained regional species pool [139]. Integrating phylogenetic *β*-diversity metrics can shed light into ecological processes (e.g., during secondary succession [141]). Null models can be further extended to account for phylogenetic relationships (i.e., Community Assembly Mechanisms by phylogenetic-bin-based null model analyses or iCamps [142]).

Considering similarities between MME and CE data can provide further avenues of innovation for analysis of *β*-diversity. For example, the analysis of functional diversity focuses on the diversity of characteristics or functional traits of diversity components [143]. Functional diversity can be estimated either through a species-centered approach, where traits are associated with a specific taxon, or estimated at the community scale through commonly used methods in microbiology [144], providing flexibility in the analysis and interpretation of MME data, which are often associated to a host (e.g., metabolomics). Similar to taxonomic and phylogenetic facets, functional diversity can be estimated through derivations of Hill numbers that can account for possible data treatments [129, 130]. For MME data derived from spectra, extant methods for deriving functional diversity estimates from remote sensing data can be applied [145].

## Conclusion: integrating MME data in multi-omics research

The future of microbiome research will likely involve combining various MME techniques (i.e., multi-omics) to determine “who is doing what” [146]. Multi-omics research may also yield new insights that link molecular biology and ecology. For example, combining metatranscriptomics and metaproteomic measurements over time has revealed that on average, archaea produce more proteins per RNA molecule than bacteria [147]. Exploring the gut microbiome of Crohn’s disease patients with both metabarcoding and metagenomics showed that metabarcoding data better predicted disease state, whereas metagenomics data were better at classifying treatment response [148]. In another study, combining metagenomic and metatranscriptomic techniques yielded novel insights into carbon cycling in soils [149].

Combining omics with other techniques can increase the specificity of the results and address complex ecological questions, providing new insights into host-microbiome interactions, revealing the trophic structure of a community, and shedding light on the metabolic pathways linking community members. For example, by combining stable isotope fingerprinting (SIF) with classic metaproteomics, direct protein-SIF, allows for the study of the individual physiology and metabolism of microbes within a community [150]. In one case, transcriptomics, metabolomics, proteomics, and metabarcoding were combined to study host-microbiome interactions in pre-diabetic individuals, revealing distinct host-microbiome responses between insulin-resistant and insulin-sensitive subjects exposed to viral infections [151].

The greatest challenge to the future of multi-omics is arguably data interpretation [152], as the analyses which inform interpretation require the integration of MME datasets which may have multiple biases. To improve interpretation, it is necessary to consider how these biases arise throughout the data generation pipeline and address them. This includes finding a consensus in experimental design (especially one that allows for different molecules to be extracted simultaneously [153, 154]), collecting necessary metadata, considering challenges in joint sampling and in storage of different molecules with different decay rates, acknowledging different coverage of reference databases [155], and explicitly selecting tools for data integration and interpretation [156]. Further research at fine spatial and temporal scales may improve the discrimination of technical noise and intrinsic variation across MME techniques and inform the development of experimental designs that minimize this noise.

From an ecological standpoint, integrating MME data begins by studying whether different MME data behave similarly across an ecological gradient of interest. As MME data interpretation becomes more advanced and ecological questions become more sophisticated, the joint analysis of multiple MME data matrices will require more advanced statistical methods. Here, statistical advances related to the fourth corner problem, which refers to the difficulty of inferring trait-environment relationships directly from environmental, species abundance, and trait data, may become instrumental [157, 158]. As analytical frameworks increase in complexity to keep up with growing needs for data integration, understanding the limitations of MME data will continue to ensure that data interpretation also improves, both in the specificity and accuracy of conclusions.

## Data Availability

Not applicable
